# Proactive inhibition is marked by differences in the pattern of motor cortex activity during movement preparation and execution

**DOI:** 10.1152/jn.00359.2021

**Published:** 2022-03-02

**Authors:** Vishal Rawji, Sachin Modi, Lorenzo Rocchi, Marjan Jahanshahi, John C. Rothwell

**Affiliations:** ^1^Department of Clinical and Movement Neurosciences, University College London Queen Square Institute of Neurology, London, United Kingdom; ^2^Department of Medical Sciences and Public Health, University of Cagliari, Cagliari, Italy

**Keywords:** dynamical systems, motor control, proactive inhibition, transcranial magnetic stimulation

## Abstract

Successful human behavior relies on the ability to flexibly alter movements depending on the context in which they are made. One such context-dependent modulation is proactive inhibition, a type of behavioral inhibition used when anticipating the need to stop or change movements. We investigated how the motor cortex might prepare and execute movements made under different contexts. We used transcranial magnetic stimulation (TMS) in different coil orientations [postero-anterior (PA) and antero-posterior (AP) flowing currents] and pulse widths (120 and 30 µs) to probe the excitability of different inputs to corticospinal neurons while participants performed two reaction time tasks: a simple reaction time task and a stop-signal task requiring proactive inhibition. We took inspiration from state space models to assess whether the pattern of motor cortex activity changed due to proactive inhibition (PA and AP neuronal circuits represent the *x* and *y* axes of a state space upon which motor cortex activity unfolds during motor preparation and execution). We found that the rise in motor cortex excitability was delayed when proactive inhibition was required. State space visualizations showed altered patterns of motor cortex activity (combined PA_120_ and AP_30_ activity) during proactive inhibition, despite adjusting for reaction time. Overall, we show that the pattern of neural activity generated by the motor cortex during movement preparation and execution is dependent upon the context under which the movement is to be made.

**NEW & NOTEWORTHY** Using directional TMS, we find that the human motor cortex flexibly changes its pattern of neural activity depending on the context in which a movement is due to be made. Interestingly, this occurs despite adjusting for reaction time. We also show that state space and dynamical systems models of movement can be noninvasively visualized in humans using TMS, thereby offering a novel method to study these powerful models in humans.

## Graphical abstract

**Figure:**
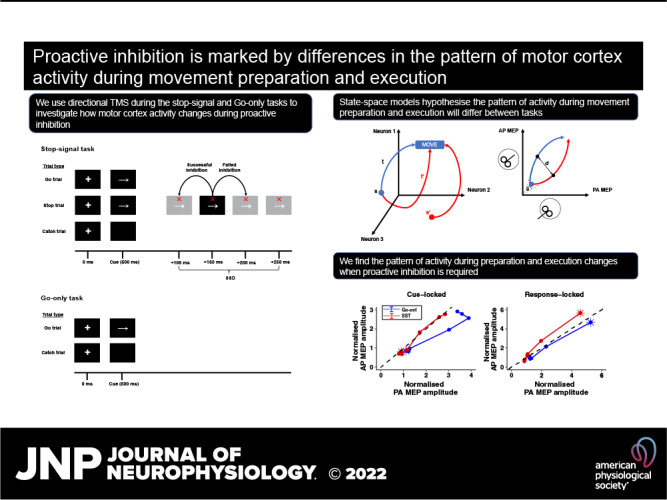

## INTRODUCTION

Imagine accelerating a car from a stationary position. The way you prepare to accelerate will be different on a quiet road compared with a road outside a school. Your motor system is capable of generating the necessary muscle forces to accelerate the car at the same speed in different contexts; around a school it is much more likely that a child will deter your path, necessitating a sudden stop in motor output. Consequently, your brain must generate the same motor output to move the car but under two very different contexts. The ability to enact this context-dependent modulation of movement is paramount to normal human functioning and in this example is called proactive inhibition—a prospective and goal-oriented type of behavioral inhibition concerned with anticipation (1, 2).

But how does the brain do this? On one hand, the motor cortex might prepare and execute movements in the same way irrespective of the context, and a separate input to the motor system may modulate or cancel the ongoing movement when required [e.g., the inhibitory hyperdirect pathway during sudden stopping (3, 4)]. More specifically, this predicts that the pattern of neural activity during movement preparation and execution will not differ across contexts. On the other hand, the motor cortex might prepare and execute movements in a fundamentally different way, based on the context in which they are to take place. In this model, the pattern of neural activity during preparation and execution will change across different contexts.

To investigate this problem, we used two behavioral tasks: a Go-only simple reaction time task that required participants to respond with button presses to a simple go cue, and a stop-signal task that had the same format as the Go-only task except that a minority of trials could be followed by a stop-signal, requiring participants to abort their response. Importantly, participants have slower responses in the latter task due to the anticipation of having to stop (proactive inhibition). Essentially, we asked participants to make the same movement (finger flexion) in different contexts: one where they know they might have to stop on some trials and another where they do not have to stop and must respond on every trial.

During the tasks, we stimulated the motor cortex with transcranial magnetic stimulation (TMS), a noninvasive brain stimulation tool that can activate underlying cortical neurons in a focal manner. If applied over M1, TMS results in muscular contractions called motor-evoked potentials (MEPs), the amplitude of which reflects excitability of the corticospinal-muscular connection. By applying TMS at different intervals during the tasks, we were able to probe motor cortex excitability during movement preparation and execution. In particular, we were interested in the “pattern” of motor cortex excitability during different phases of movement, described in the next paragraph. To this end, we applied TMS in two different coil orientations [postero-anterior (PA) and antero-posterior (AP) flowing current], which are known to activate two (largely separate) populations of cortical motor neurons that have a common output (5–7). Recent work has expanded on this distinction, finding that altering TMS pulse width (120 and 30 µs) can further differentiate PA and AP neuronal inputs (8–11). PA and AP inputs activate separate cortical circuits (12, 13) and are behaviorally separable, given that they are differentially modulated during movement preparation (14), behavioral plasticity (15), and motor learning (16).

**Figure 1.**
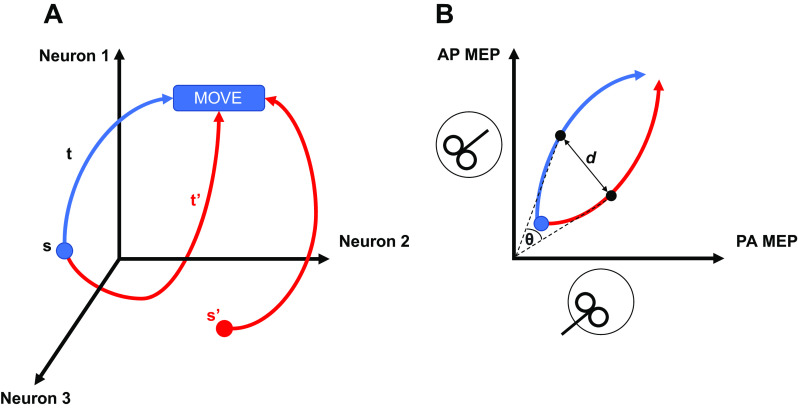
Neural activity during proactive inhibition visualized through state space models. *A*: dynamical systems models posit that neural activity exists in a multidimensional space with each neuron’s activity representing one axis of this space. The figure shows a three-dimensional space made up of the activity of three individual neurons. Activity of each of the neurons at any point in time can be plotted in this space. At the end of preparation, population activity occupies a subspace of this overall space, denoted by the blue circle (s). Upon the receipt of a trigger to move, population activity then evolves into the movement along a trajectory (t). Proactive inhibition might be employed by varying the trajectory of the movement (t’) or by setting a different initial state (s’) at the end of preparation and before the receipt of the trigger to move. *B*: this idea can be modelled as activities in PA and AP networks measured by their corresponding motor-evoked potentials (MEPs) during movement preparation and execution. The similarity/difference between trajectories can be measured using distance metrics. Two measures can be used: the distance between two trajectories at a given time point (Euclidean distance – *d*); or the angle between the two vectors drawn from the origin to each of the two points (cosine distance – θ). AP, antero-posterior; PA, postero-anterior.

Patterns of neural activity are typically visualized using state space models that treat each neuron’s activity as an individual axis in multidimensional space (Fig. 1*A*). A point in this space determines the state of neural population activity at a particular time. By plotting these points throughout time, a trajectory is drawn, which determines the change of neural population state across time (17). These states and trajectories reflect important features of movement dynamics and behavior such as parsing motor preparation and execution into two discrete processes with independent, putative dynamics. Inspired by this, we sought to use TMS to visualize corticospinal excitability through the state space framework. PA and AP inputs activate separate cortical inputs to a common motor output; we therefore treated their respective activities as *x* and *y* dimensions on a two-dimensional (2-D) plane that represents the dimensions of a state space upon which motor cortex activity unfolds during motor preparation and execution (Fig. 1*B*). Specifically, the pattern of motor cortex excitability would be manifested as the activities in PA_120_ and AP_30_ networks.

## METHODS

### Participants

Sixteen healthy volunteers (9 males, 16 right-handed) aged 19–33 yr (mean age: 24.65, SD: 4.13) participated. The study was approved by the UCL Ethics Committee and written informed consent was obtained from all participants. The study was performed in accordance with the Declaration of Helsinki. None of the participants had contraindications to TMS, which was assessed by a TMS-screening questionnaire based on the one published by Keel et al. (18).

### Electromyography Recordings

Throughout the experiment, participants were seated comfortably in a nonreclining chair, with their right index finger rested over the “M” key on a keyboard. Their forearms were supported using a cushion. Electromyographic (EMG) activity was recorded from the right first dorsal interosseous (FDI) muscle using 19 × 38 mm surface electrodes (Ambu WhiteSensor 40713) arranged in a belly-tendon montage, with a sensor area of 77 mm^2^. The raw signals were amplified, and a bandpass filter was also applied (20 Hz to 2 kHz, Digitimer, Welwyn Garden City, UK). Signals were digitized at 5 kHz (CED Power 1401; Cambridge Electronic Design, Cambridge, UK), and data were stored on a computer for offline analysis (Signal v. 5.10, Cambridge Electronic Design, UK).

### Transcranial Magnetic Stimulation

MEPs in the right FDI muscle were evoked using a controllable TMS (cTMS) device (cTMS3, Rogue Research Inc., Canada), connected to a standard figure-of-eight coil (wing diameter 70 mm, Magstim, United Kingdom). The hotspot was identified as the area on the scalp where the largest and most stable MEPs could be obtained for the right FDI muscle, using a suprathreshold TMS pulse. The hotspot was marked on the participant’s scalp using a colored pencil that was removed after the experiment had concluded. Importantly, hotspots were found separately for PA and AP coil orientations since they have distinct anatomical bases. We delivered monophasic TMS pulses in two ways. With the coil held approximately perpendicular to the presumed central sulcus and tangentially to the skull, TMS was given either with the coil handle pointing backward for PA stimulation at 120 µs pulse width (PA_120_) or with the coil handle pointing forward for AP stimulation at 30 µs pulse width (AP_30_).

### Stop-Signal Task and Go-Only Simple Reaction Time Task

**Figure 2.**
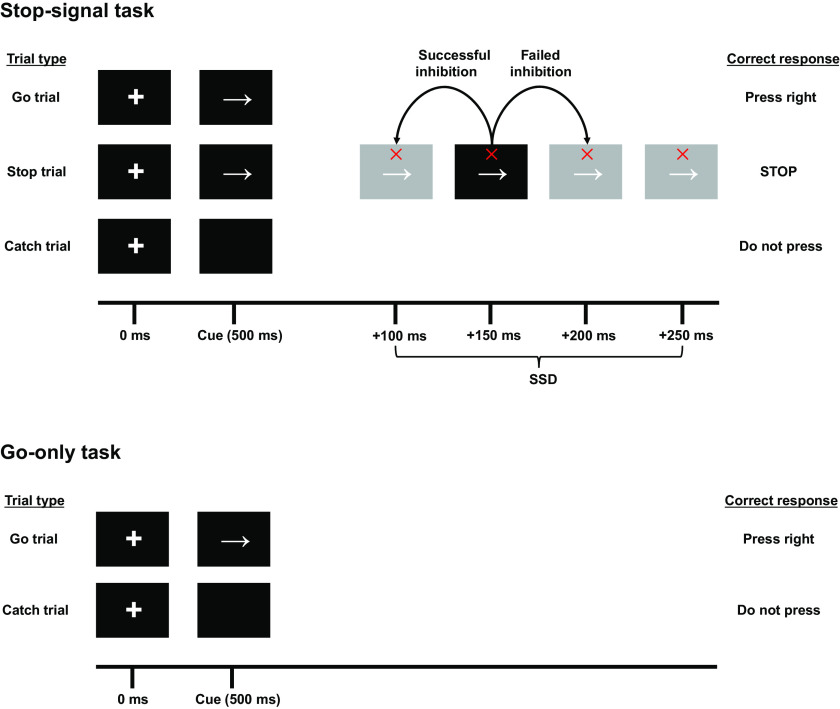
The stop-signal task (SST) and Go-only tasks. SST: go trials consisted of presentation of a fixation cross, followed by a go cue (*right* arrow) 500 ms later. In 25% of trials, the right arrow was followed by a stop-signal (red cross) at one of four SSDs (100, 150, 200, or 250 ms after the arrow). Participants attempted to abort their button press on presentation of a stop-signal. Failure to do so resulted in the next stop-signal having a shorter SSD (−50 ms) whereas successful stopping led to the next SSD becoming longer (+50 ms). PA_120_ or AP_30_ TMS was delivered on go trials at one of seven time points (counterbalanced and randomized), or 1,000 ms after presentation of the fixation cross (white cross) where no signals are shown (baseline trial). Go-only task: comprised of go and catch trials only; TMS was delivered at the same time points described earlier. AP, antero-posterior; PA, postero-anterior; SSD, stop signal delay; TMS, transcranial magnetic stimulation.

Participants were asked to perform two blocks of the stop-signal task (SST) and two blocks of a simple reaction time (Go-only) task, which were driven by custom-made MATLAB (the MathWorks) scripts using Psychtoolbox (19). In the Go-only task (Fig. 2), trials began with the presentation of a white fixation cross on a black background. A go cue (right arrow) was presented 500 ms later, which instructed participants to press the “M” key on the keyboard as fast as possible with their right index finger (go trial, *n* = 105). On fifteen trials, only a fixation cross was displayed. These served as catch trials and were randomly presented throughout the block. In essence, the Go-only task was a simple reaction time task that required less proactive control than the SST. For the SST (Fig. 2), go (*n* = 105) and catch (*n* = 15) trials were presented as in the Go-only task. However, the SST included stop trials (*n* = 35), whereby a stop signal (red cross) appeared above the go cue at a variable delay after the go cue, instructing participants to abort their motor responses. This variable delay is known as the stop signal delay (SSD) and can range from 100 to 250 ms in 50 ms time steps (100, 150, 200, and 250 ms). Changing the SSD changes the difficulty of stopping when a stop signal is shown: short SSDs are easy to stop to, whereas longer SSDs make stopping more difficult. The SSD was initially set at 150 ms and changed on a trial-by-trial basis, depending on the outcome of the previous stop trial (dynamic tracking algorithm): if the participant successfully prevented their button press on a stop trial, the next stop trial would have its SSD set 50 ms later, whereas if the participant failed to stop, the next stop trial would have its SSD set 50 ms earlier (20–22). The dynamic tracking algorithm has been shown to reliably induce a convergence onto 50% successful inhibition across participants, and hence, ensures similar task performance across participants. Effectively, participants were responding on go trials as in the Go-only task, except with the prior knowledge that they might have to stop in anticipation of a stop signal—they were responding with restraint (23) and employing proactive inhibition. The order of trials was pseudorandomized, such that one in every four trials contained a stop signal. Intertrial interval was set to 1,750 ms.

The main behavioral measure of interest was the response delay effect (RDE)—a reaction time measure of proactive inhibition. This was calculated as the difference in reaction time on go trials in the SST and Go-only task. Other behavioral measures collected included go reaction time (reaction time on go trials), stop respond reaction time (reaction time on failed stop trials), average SSD, and p(inhibit) (proportion of correct stop trials on the SST). We also calculated the stop-signal reaction time (SSRT) using the mean method (21) (mean go reaction time − mean SSD). The SSRT is a measure of reactive inhibition.

### Integration of TMS with the Stop-Signal and Go-Only Simple Reaction Time Tasks

TMS was given on all trials, in all blocks, to the M1 representation for the right FDI muscle, at an intensity required to produce a test MEP of 0.5 mV peak-to-peak amplitude; a test stimulus of 0.5 mV was chosen to limit the effect of TMS on reaction time, while still being able to capture the dynamic range of responses during movement. During go trials, one TMS pulse was given randomly at one of seven time points (at the go cue and 50, 100, 150, 200, 250, and 300 ms after the go cue). Fifteen MEPs were taken at each time point. In the 15 baseline trials, TMS was given 1,000 ms after presentation of the fixation cross (white cross) to assess corticospinal excitability (CSE) at rest.

### Experimental Protocol

Participants were first shown the two types of task that they would need to complete. They then performed a truncated version of each block so that they understood/learned the task before the collection of data used in this study. The order of task and TMS combination was randomized using a random number generator, after which participants completed each block in turn. Breaks were permitted between blocks.

### Data Analysis

Data handling and analyses were performed using custom-made scripts in MATLAB (MathWorks, v. 2017a). We used the lme4 package (24) in R (v. 1.1.463) to run linear mixed models and post hoc *t* tests. All statistics were conducted as per a within-subject design given that the simulated Go-only and SST versus Go-only trajectories were derived from the same participants. Data normality was assessed by visualizing QQ-plots of residuals. Where data deviated from normality, log-transformations were applied to dependent variables before statistical analyses.

Trials with reaction times exceeding 1,000 ms were classed as omission errors due to lapses in concentration. The magnitude of proactive inhibition was determined as the reaction time difference on go trials between the SST and Go-only task, also known as the RDE. We used a linear mixed model with COIL ORIENTATION (PA_120_, AP_30_) and TASK (SST, Go-only) as fixed effects and modeled participant identity as a random intercept effect. Post hoc paired *t* tests with Tukey’s correction were used to interrogate significant interactions.

MEPs were preprocessed using visual inspection. Trials where TMS arrived during or after the EMG burst were excluded from analysis. We used a linear mixed model to assess how CSE changed after presentation of the go cue, for different coil orientations and under different tasks. For the cue-locked analysis, COIL ORIENTATION (PA_120_, AP_30_), TASK (SST, Go-only), and TIME POINT (Cue, 50, 100, 150, 200, 250, and 300 ms) were treated as fixed effects, whereas the participant identity was chosen as a random intercept effect. We also represented CSE between stopping conditions and inputs from the viewpoint of movement execution. To do this, we calculated the time between TMS delivery and response, then grouped MEPs according to 50 ms time bins from the response (300–350, 250–300, 200–250, 150–200, 100–150, and 50–100 ms before movement). Similar to the cue-locked analysis outlined earlier, we performed a linear mixed model with the same factors as above, except that the factor TIME POINT represented time periods preceding the response (300–350, 250–300, 200–250, 150–200, 100–150, and 50–100 ms before movement). We included all interactions between our main factors in our models given that we were interested in each of their effects. Tukey corrected post hoc paired *t* tests were used to further investigate any significant interaction effects from the linear mixed models.

We took inspiration from state space modeling to visualize the pattern of neural activity during movement preparation and execution in the SST and Go-only tasks. To do so, we treated PA_120_ and AP_30_ inputs as dimensions on a 2-D plane by plotting normalized to baseline PA_120_ MEPs (*x* axis) against normalized to baseline AP_30_ MEPs (*y* axis). Points on the plot show PA_120_ and AP_30_ MEPs at each time point for cue-locked (Cue, 50, 100, 150, 200, 250, and 300 ms) and response-locked (300–350, 250–300, 200–250, 150–200, 100–150, and 50–100 ms before movement) analyses. By treating each point in space as a vector (*x* coordinate = normalized PA_120_ amplitude, *y* coordinate = normalized AP_30_ amplitude), we calculated two measures of distance to help further understand the differences between trajectories in Go-only and SST conditions. Euclidean distances are sensitive to the magnitude of each vector, whereas cosine distances are insensitive to magnitude and quantify distance in terms of the difference in angles from the origin between two vectors. In brief, Euclidean distances signify the differences in the magnitude, whereas cosine distances represent difference in the relative weighting of PA_120_/AP_30_ inputs. We used a method described by Ames et al. (25) to test whether the trajectories taken differed significantly when proactive inhibition was used in the SST. First, simulated trajectories were first drawn (via bootstrapping with replacement) from the Go-only task. Each of these trajectories represent the neural trajectories that would occur by chance if there was no effect of proactive inhibition. Next, the distance between the simulated Go-only and original Go-only trajectories were then calculated for each time point. These distances represent the distances if there was no effect of proactive inhibition. Finally, we statistically compared the simulated distances with the distances calculated between the SST and Go-only task using a linear mixed model for each time point. Fixed effects in the linear mixed model included COIL ORIENTATION TIME and ANALYSIS TYPE (real, simulated), with participant identity modeled as a random intercept effect.

## RESULTS

### Physiological Measurements

No significant differences were found between the amplitudes of the baseline MEPs across sessions or between PA_120_ and AP_30_ conditions. As expected, AP_30_ TMS test stimulus (mean: 82.0%, SD: 10.0% of maximum stimulator output) intensities were higher than those for PA_120_ (mean: 29.6%, SD: 3.1% of maximum stimulator output) stimulation. Baseline MEPs could not be elicited for three participants. Consequently, 16 participants provided data for PA_120_ TMS and 13 for AP_30_ TMS. We note that the differences in stimulator intensities used between PA_120_ and AP_30_ conditions cannot be interpreted as absolute differences given that maximum stimulator output varies as a function of pulse width.

### Behavioral Measures

Behavioral measurements in the SST and Go-only simple reaction time task are shown in Table 1. In the SST, the dynamic tracking algorithm correctly resulted in a convergence of successful inhibition in ∼50% of trials. The linear mixed model showed significant effects of COIL ORIENTATION [*P* = 0.028, *F*(1,44.49) = 5.21] and TASK [*P* < 0.001, *F*(1,40.74) = 79.85] but no significant interaction [*P* = 0.224, *F*(1,40.74) = 1.52]. This meant that reaction times were slightly slower for AP_30_ TMS trials than PA_120_ trials, and faster in the Go-only task than SST. This reaction time difference due to anticipatory slowing (RDE) is the behavioral manifestation of proactive inhibition.

**Table 1.** Behavioral measurements from the SST and Go-only simple reaction time tasks

		SST		Go-Only	
Measure	Measure Description	PA_120_	AP_30_	PA_120_	AP_30_
Go reaction time	RT to go stimulus in the critical direction	391.55 (35.01)	402.36 (44.42)	288.31 (32.12)	324.15 (52.28)
P(inhibit)	% correct inhibition	50.54 (7.36)	56.70 (11.30)		
Stop respond	RT on failed stop trials	287.84 (33.13)	319.69 (47.90)		
Go omission	% of omissions	0.36 (0.68)	0.44 (0.84)	0.36 (0.84)	0.66 (0.98)
Stop signal delay	Delay between go and stop trials	167.05 (25.42)	185.29 (31.52)		
SSRT	Calculated time to abort response	224.50 (27.75)	216.98 (32.59)		

RT, reaction time; SSRT, stop-signal reaction time; SST, stop-signal task.

The table shows the behavioral measures from the SST, Go-only simple reaction time task. Measures are accompanied by SD in brackets. Reaction times are given in milliseconds. SSRT is stop signal reaction time.

Evolution of corticospinal excitability in stop-signal and Go-only simple reaction time tasks.

#### Preparation of movement: cue-locked analysis.

**Figure 3.**
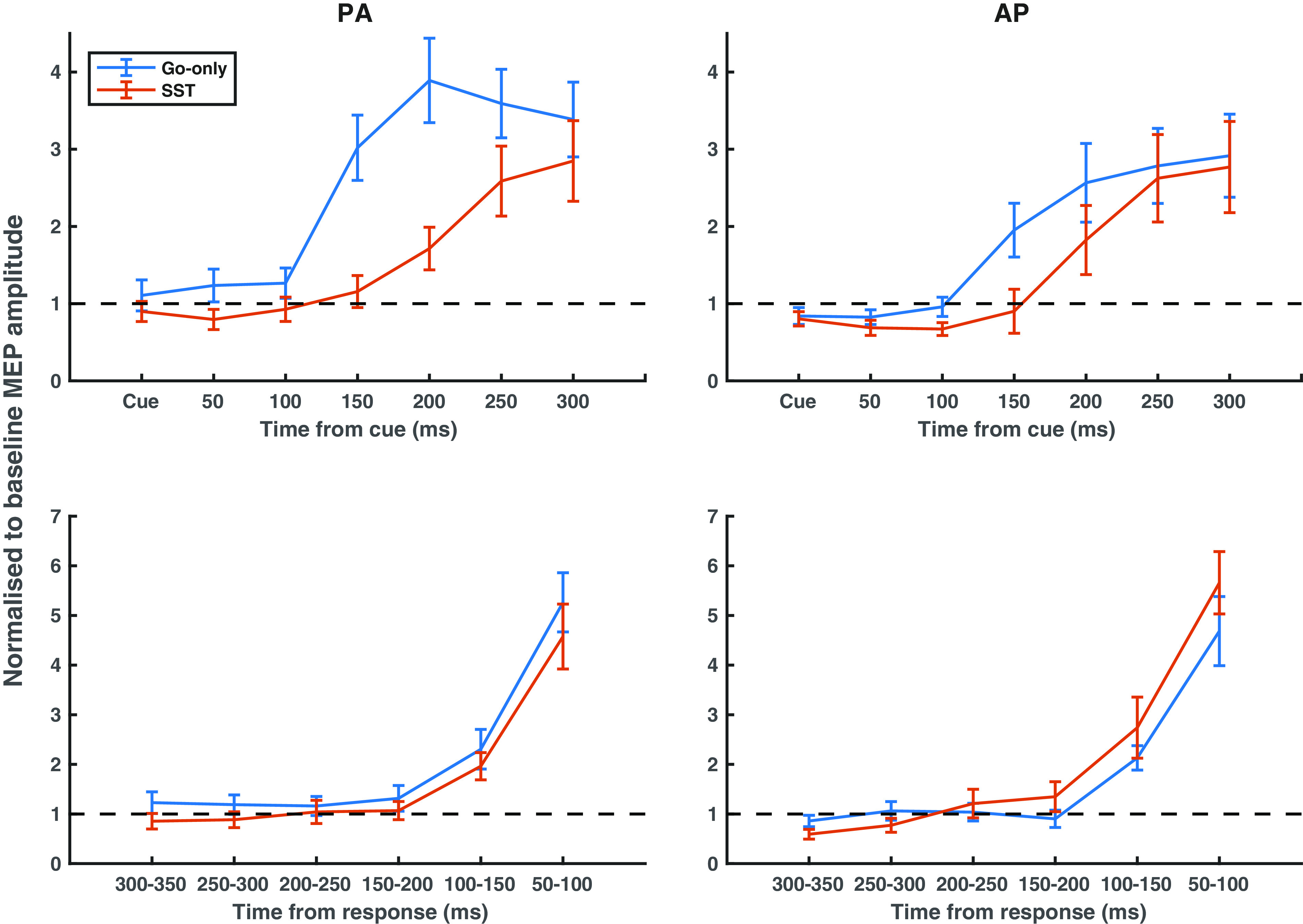
Corticospinal excitability changes during the stop-signal task (SST) and Go-only task for AP_30_ and PA_120_ TMS. *Top row*: motor-evoked potentials (MEPs) are taken on go trials at various times after the go cue has been presented, for the Go-only simple reaction time task and SST. *Bottom row*: MEPs are sorted into 50 ms bins before the response. MEP values are normalized to baseline MEP value. Graphs represent responses evoked using PA_120_ TMS (*left column*) and AP_30_ TMS (*right column*). Error bars represent means ± SE. Go-only task: blue line, SST: red line. AP, antero-posterior; PA, postero-anterior.

The SST was used to probe the temporal dynamics of CSE changes during which proactive inhibition is implemented (26). This was compared with the same TMS timings in a task where less proactive inhibition should be employed during the Go-only simple reaction time task. A linear mixed model showed significant effects for COIL ORIENTATION [*P* < 0.001, *F*(1,324) = 14.66], TASK [*P* < 0.001, *F*(1,324) = 48.30], and TIME POINT [*P* < 0.001, *F*(6,324) = 53.71]. Of note, there was a significant TASK × TIME POINT [*P* < 0.001, *F*(6,324) = 4.44] interaction. We used post hoc *t* tests to investigate this interaction (full interaction comparisons are shown in the Supplemental Material; all Supplemental material is available at https://doi.org/10.6084/m9.figshare.19193396.v1). On go trials within the SST, the main rise in excitability, indexed by the timepoint at which CSE became significantly greater than CSE at the cue, occurred later than on Go-only trials for both (Go-only: 150 ms, *P* < 0.001, *t* = 6.19; SST: 200 ms, *P* = 0.002, *t* = 4.29). These results are summarized by plots in the top row of Fig. 3.

#### Execution of movement: response-locked analysis.

To assess CSE from the perspective of movement execution, we realigned the data to the time of the response onset, thereby performing a response-locked analysis (Fig. 3, *bottom row*). A linear mixed model did not find any statistically significant effects of COIL ORIENTATION [*P* = 0.272, *F*(1,266.05) = 1.21] or TASK [*P* = 0.149, *F*(1,266.04) = 2.10] and as expected revealed a significant effect of TIME [*P* < 0.001, *F*(5,266.06) = 92.98]. There were no statistically significant interactions. From this, it appears that CSE preceding a response did not differ between coil orientations or task.

### Assessing the Pattern of Neural Activity during Movement Preparation and Execution

We next assessed if the pattern of neural activity during movement preparation and execution changed as a function of proactive inhibition. An important distinction between the previous analysis and the subsequent one is how the factor COIL ORIENTATION is considered: in the former, there are two groups (PA_120_ and AP_30_) with corresponding CSEs for each group, which allows for the effect of COIL ORIENTATION to be assessed. However, patterns of neural activity are assessed by considering M1 CSE as the combination of PA_120_ and AP_30_ inputs, thereby compressing the factor COIL ORIENTATION into one group. This means that each value/dependent variable becomes a vector with the first value being PA_120_ MEP amplitude and the second value being AP_30_ MEP amplitude. Accordingly, this vector-based analysis requires a different analytical approach than that described in the previous section.

**Figure 4.**
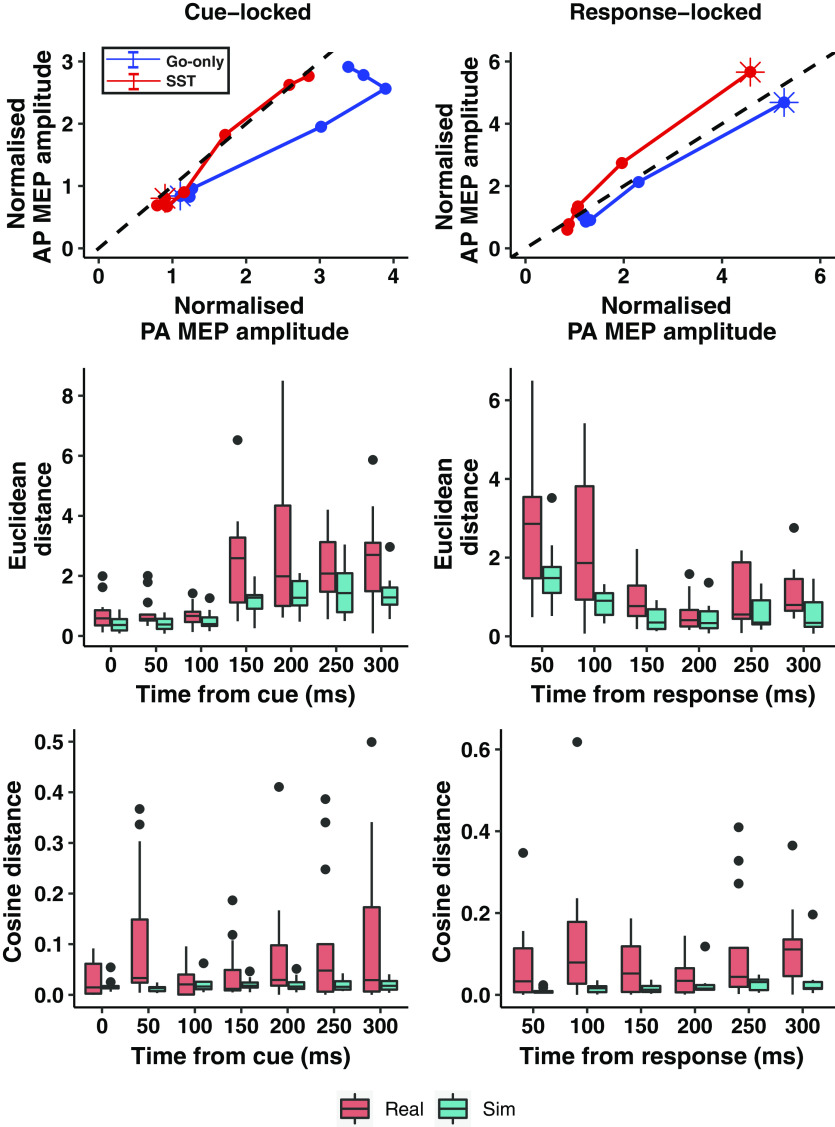
Motor cortex population-level activity during movement preparation and execution. *Top row*: motor cortex population-level activity is represented as a combination between PA_120_ and AP_30_ inputs. Each plot shows the trajectory taken by this population activity throughout movement. *Left*: cue-locked analysis: activity starts at the cue (shown by stars). Activity then progresses over time, with each marker (circle) representing a time-point (Cue, 50, 100, 150, 200, 250, and 300 ms). This is shown for the SST (red line) and Go-only task (blue line). *Right*: response-locked analysis: stars represent population activity 50–100 ms before movement. Working backward, time-points are as shown in the bottom row for Fig. 3. Dashed, *x* = *y* lines represent balanced PA_120_ and AP_30_ CSE. Euclidian (*middle row*) and cosine (*bottom row*) distances are shown for our data and from bootstrapped simulated data neural trajectories drawn from the same distribution as Go-only data. Smaller values indicate greater similarity between corresponding time points. AP, antero-posterior; MEP, motor-evoked potential; PA, postero-anterior; SST, stop-signal task.

We plotted normalized to baseline PA_120_ and AP_30_ MEPs against each other for each timepoint in the cue and response-locked analyses (Fig. 4, *top row*). The resultant trajectories show how population-level activity within M1 evolves during movement preparation and execution. The cue-locked analysis shows that M1 population activity evolves within the same subspace early after cue presentation (*bottom left*). Approximately 150 ms later, activity increases in both neuronal inputs and occupies a separate space at the end of movement (*top right*). Notably, the trajectories taken varied between the tasks given significant differences in Euclidean distances {TIME: [*P* < 0.001, *F*(6,156) = 42.14]; ANALYSIS TYPE: [*P* < 0.001, *F*(1,156) = 45.89; TIME × ANALYSIS TYPE: [*P* = 0.741, *F*(6,156) = 0.59]}. Cosine distances did not significantly differ by ANALYSIS TYPE [*P* = 0.466, *F*(1,156) = 0.53], TIME [*P* = 0.22, *F*(6,156) = 1.39], and there was no interaction [*P* = 0.100, *F*(6,156) = 1.81].

The response-locked analyses similarly showed a difference in trajectories, despite correcting for reaction time. We found significant differences in Euclidean distances when proactive inhibition was required [*P* < 0.001, *F*(1,116.26) = 29.06] and varied significantly over time [*P* < 0.001, *F*(5,116.69) = 18.67], without a significant interaction [*P* = 0.419, *F*(1,116.28) = 1.00]. A significant difference in cosine distance was seen in the SST [*P* = 0.007, *F*(1,116.87) = 7.56] but there was no significant effect of time nor interaction. It appears that when viewing the patterns of neural activity, behaviorally equivalent responses are prepared and executed differently by M1 population activity, dependent on the requirements for proactive inhibition.

## DISCUSSION

We sought to understand how M1 would prepare and execute the same movement when made under different contexts. More specifically, we used two reaction time tasks requiring an identical response, which differed in their task instructions, extent of motor preparation, and anticipatory slowing, such that participants employed greater proactive inhibition in the SST. By applying TMS, we observed that, relative to the time of onset of the go cue, CSE increased later during the go trials of the SST compared with the Go-only task (Fig. 3, cue-locked analysis). From the viewpoint of motor execution (Fig. 3, response-locked analysis), the rate of increase in CSE was the same in both tasks. Overall, this suggests that proactive inhibition delays the initial rise in CSE after a cue is presented, rather than causing a slower rise of CSE to a notional “threshold” for movement (27) – a feature also found when macaque monkeys delay saccades during a visual SST (28). We placed particular interest on how patterns of neural activity might be differentially represented during proactive inhibition, and used state space models to visualize this. In doing so, we found that proactive inhibition was marked by a difference in the trajectories taken preceding movement and by the relative weighting in PA_120_ and AP_30_ inputs (Fig. 4).

### A Dynamical Systems View of Proactive Inhibition

The dynamical systems view of motor control is becoming a popular way in which to visualize neural activity during movement (17, 29). This proposes that, instead of representing explicit features of the movement (such as direction or velocity), M1 activity during motor preparation sets the initial state of a dynamical system, that evolves into the desired movement (30) upon the receipt of some trigger to move (31). Consequently, neural activity during movement preparation and execution reflects the transition from one state to the next under some dynamical rule, and hence not all M1 activity need represent movement-related activity. Crucially, the dynamical system arises as an interplay between populations of neurons during motor preparation and execution and is not appreciated from the single-neuron perspective, which has traditionally driven theories of motor control.

The dynamical systems view uses state space models to visualize population-level neural activity. While largely successful, the investigation of dynamical systems in humans has been limited by the difficulty and infeasibility of large-scale single-neuron recordings. A recent study has attempted to overcome this by using an innovative experimental design to show that choice traces similar to those seen in nonhuman primates using dynamical systems models (32) can be visualized in premotor cortex using magnetoencephalography (33). The current study similarly shows that M1 activity can also be interpreted in a dynamical systems framework using TMS. The dynamical systems approach is appropriate to interpret TMS results, given that they both rely on population-level activity. By plotting PA_120_ CSE against AP_30_ CSE, we visualize how M1 “population” activity evolves throughout time and through movement preparation and execution (Fig. 4). Akin to the findings using a dynamical systems approach, we see that activity during movement preparation evolves in a particular, confined subspace for ∼150 ms after cue presentation. That is, activity occupies the bottom left of the cue-locked plot for 150 ms (similarly, activity occupies the bottom left of the response-locked plot 300–350 ms before movement). Following this, M1 population CSE shows a large increase upon receipt of a trigger for movement execution, to a different area in the subspace (Fig. 4, *top right* of the cue-locked and response-locked plots).

An important observation is that the trajectory taken (Fig. 4) during movement differs between tasks. More specifically, the absolute magnitude (Euclidean distance) and balance within PA_120_ and AP_30_ inputs (cosine distance) differ when proactive inhibition is called upon. Overall, visualization through this framework shows that the pattern of neural activity within M1 differs depending on the context in which the movement is due to be made, a feature not apparent from conventional analyses as in Fig. 3.

### Context-Dependent Modulation of Movement

The introduction outlined that context-dependent modulation of movement might be implemented in a number of ways: in one, the motor command output by M1 is the same irrespective of the context, and context-dependent modulation of movement comes about by modulation of this descending output. In another, M1 might change its descending motor command depending on the context. The present study provides evidence for the latter hypothesis, showing that the pattern of neural activity within M1 is dependent on the context in which that movement will be performed. But how does the brain generate this context-relevant activity? In dynamical systems models of movement, neural activity occupies a state at the end of preparation that is relevant to the upcoming movement, which then evolves into the movement upon receipt of some trigger. Context-dependent modulation of movement may arise secondary to: *1*) setting of an alternative preparatory state (34, 35), *2*) difference in the trigger that causes evolution of preparatory to movement activity, or *3*) both.

The ability to shift the distribution of CSE between neuronal inputs may allow for qualitatively equivalent movements to be performed in a variety of ways depending on task-specific goals (36). In fact, a recent study by Baudry and Duchateau (37) has shown that CSE rise time before EMG onset occurs 100 ms earlier for smooth ramp than ballistic contractions of tibialis anterior (akin to the response-locked analysis in the present study). They proposed that the difference in rise time was related to differences in how short-interval intracortical inhibition (SICI) changed before EMG onset: a sharp decrease in SICI was observed 200–100 ms before EMG onset for ballistic contractions, whereas SICI decreased smoothly over time for ramp contractions (37). This suggests that intracortical dynamics can be flexibly adapted depending on how the movement is made. The present study adds to this idea and is the first visualization of CSE during movement preparation and execution represented as the interplay between different neuronal inputs in humans depending on movement context.

### Limitations

The pseudorandom design of the task meant that participants could develop expectancy and learn to anticipate the stop signal, which could potentially confound measures of response inhibition. However, we observed that participants successfully inhibited their responses on ∼50% of stop trials, showing that participants correctly engaged with task demands.

In light of the equivalence shown by the response-locked analyses, we concluded that participants were delaying their trigger to move. Variants of the SST have shown that proactive inhibition can sometimes be mediated by alterations in the threshold before which movement is triggered (26, 27). Given these apparent differences in the strategy used to mediate proactive inhibition, it may be the case that our findings are a feature of task design and differ when proactive inhibition is mediated differently. The movement (right finger button press) was known throughout the experiment and did not change, meaning that the same movement was prepared in both tasks, on all trials. Consequently, the similarity in response-locked CSE profiles (Fig. 3) may be so because the movement to be prepared is the same in both tasks (although this would not account for differences in the population-activity analysis in Fig. 4). Future studies should aim to change the way in which the same movement is prepared to help establish whether movement execution CSE is dependent on motor preparation.

Button presses involve flexion of the metacarpophalangeal joint in which FDI is a synergist with flexor indicis profundus (38). We chose to record from FDI for two reasons: first, it has a lower threshold to TMS, making it a more pleasant experience for participants, and second, because it is easier to isolate the activity of FDI with surface EMG than the deep flexor muscle. However, it would have been useful to have additional confirmation of the results from other muscles involved in the movement. Nevertheless, previous studies have shown FDI CSE modulation during movement preparation and execution of button presses (39–41).

We noted a statistically significant effect of coil orientation on reaction time; on average, reaction times for AP_30_ TMS were 11 ms longer in the Go-only task and 36 ms longer in the SST. Although this would not affect the results of the cue-locked analyses (as MEPs were aligned to the cue), they could confound the response-locked analysis, since MEPs were aligned to the response. The difference in reaction time due to coil orientation has been difficult to resolve given the interactions of pulse width and TMS intensity with the need to evoke the same amplitude MEP for both orientations. Nevertheless, we think that the effect of reaction time prolongation on our results may be limited since bin widths were 50 ms wide, larger than the reaction time differences observed.

### Conclusions

We set out to investigate how the motor cortex would prepare and execute equivalent movement when made under different contexts. When proactive inhibition was required, movements were prepared by delaying the rise in CSE. Using state space models and directional TMS, we show that proactive inhibition might operate by altering the pattern of activity used by M1 to execute a movement, indexed by different trajectories before movement onset or setting of an alternative initial state before the rise in CSE leading up to movement.

## DATA AVAILABILITY

The data and code used to produce these findings are posted at https://github.com/vishrawji/Proactive-inhibition-is-marked-by-differences-in-the-pattern-of-motor-cortex-activity-during-movemen.

## SUPPLEMENTAL DATA

10.6084/m9.figshare.19193396.v1 Supplemental Material: https://doi.org/10.6084/m9.figshare.19193396.v1.

## GRANTS

This study was funded by doctoral training grant MR/K501268/1 from the Medical Research Council.

## DISCLOSURES

No conflicts of interest, financial or otherwise, are declared by the authors.

## AUTHOR CONTRIBUTIONS

V.R., M.J., and J.C. R. conceived and designed research; V.R. and S.M. performed experiments; V.R. analyzed data; V.R., L.R., M.J., and J.C.R. interpreted results of experiments; V.R. prepared figures; V.R. drafted manuscript; V.R., S.M., L.R., M.J., and J.C.R. edited and revised manuscript; V.R., S.M., L.R., M.J., and J.C.R. approved final version of manuscript.

## ENDNOTE

At the request of the authors, readers are herein alerted to the fact that additional materials related to this manuscript may be found at https://github.com/vishrawji/Proactive-inhibition-is-marked-by-differences-in-the-pattern-of-motor-cortex-activity-during-movemen. These materials are not a part of this manuscript and have not undergone peer review by the American Physiological Society (APS). APS and the journal editors take no responsibility for these materials, for the website address, or for any links to or from it.
